# The Bidirectional Link Between Diabetes and Kidney Disease: Mechanisms and Management

**DOI:** 10.7759/cureus.45615

**Published:** 2023-09-20

**Authors:** Mahendra Kumar, Shah Dev, Muhammad Usman Khalid, Sowmya Manjari Siddenthi, Muhammad Noman, Chris John, Chiderah Akubuiro, Anum Haider, Riya Rani, Maham Kashif, Giustino Varrassi, Mahima Khatri, Satesh Kumar, Tamam Mohamad

**Affiliations:** 1 Medicine, Sardar Patel Medical College, Bikaner, IND; 2 Internal Medicine, Jinnah Sindh Medical University, Karachi, PAK; 3 Medicine, King Edward Medical University, Lahore, PAK; 4 Internal Medicine, Osmania Medical College, Hyderabad, IND; 5 Internal Medicine, Ziauddin University, Karachi, PAK; 6 Internal Medicine, University College Dublin, Dublin, IRL; 7 Medicine, Trinity Medical Sciences University, Ribishi, VCT; 8 Internal Medicine, Bahria University Medical and Dental College, Karachi, PAK; 9 Internal Medicine, Peoples University of Medical and Health Sciences for Women, Nawabshah, PAK; 10 Medicine, Khawaja Muhammad Safdar Medical College, Sialkot, PAK; 11 Pain Medicine, Paolo Procacci Foundation, Rome, ITA; 12 Medicine and Surgery, Dow University of Health Sciences, Karachi, PAK; 13 Medicine and Surgery, Shaheed Mohtarma Benazir Bhutto Medical College, Karachi, PAK; 14 Cardiovascular Medicine, Wayne State University, Detroit, USA

**Keywords:** management, dkd, disease, kidney, diabetes

## Abstract

The complex and mutually influential connection between diabetes mellitus and chronic kidney disease (CKD) is a significant focal point in the current healthcare landscape. Diabetes, a medical condition characterized by elevated blood glucose levels resulting from impaired insulin action or secretion, has become a significant global epidemic. It poses considerable challenges to healthcare systems and affects millions of individuals worldwide. Concurrently, CKD, characterized by the gradual decline of kidney function, has become a persistent health challenge. This narrative review explores the complex relationship between these two conditions, shedding light on their significant implications for public health, clinical practice, and biomedical research. The correlation between diabetes and kidney disease is not merely coincidental. Diabetes is recognized as a significant risk factor for CKD, as individuals with diabetes are considerably more vulnerable to developing renal complications. Diabetic nephropathy, a distinct type of kidney disease closely associated with diabetes, is a significant factor in developing end-stage renal disease. It is imperative to implement efficient diabetes management strategies to regulate blood sugar levels and prevent potential kidney damage. On the other hand, kidney disease may contribute to the development of diabetes. The kidneys regulate glucose levels by filtering the blood and selectively reabsorbing glucose as necessary. In compromised kidney function, such as CKD, impaired glucose metabolism can give rise to insulin resistance and diabetes. As a result, the management of kidney disease plays a dual role in both preserving renal function and preventing diabetes in individuals who are at risk. The coexistence of diabetes and kidney disease in a patient presents complex clinical challenges. Achieving effective management requires a meticulous balance between glycemic control and preservation of renal function. Failing to maintain this delicate equilibrium can lead to cardiovascular complications and subsequent hospitalizations. This comprehensive narrative review aims to thoroughly examine the pathophysiological mechanisms that connect diabetes and kidney disease. It will provide insights into the clinical manifestations and diagnostic methods, explore various approaches to managing the condition, discuss the crucial role of nutrition, delve into pharmacological interventions, emphasize the importance of patient education and self-care, and shed light on emerging research areas. In addition to impacting individual health outcomes, this reciprocal relationship has significant implications for healthcare systems, socioeconomic landscapes, and public health policy. Comprehending this complex interaction is crucial for making well-informed clinical judgments, improving patient care, and developing a more efficient public health approach to address the interconnected issues of diabetes and kidney disease.

## Introduction and background

The convergence of diabetes mellitus and kidney disease entails a multifaceted and intricate interaction within contemporary medicine, with significant implications for public health, clinical practice, and biomedical research. Diabetes mellitus is a metabolic disorder characterized by hyperglycemia due to impaired insulin secretion or action. This condition has reached epidemic proportions worldwide [1]. Over the past few decades, the increasing prevalence of this issue has elevated its status to that of a significant public health concern. It has profoundly impacted the lives of millions and presents a considerable challenge to healthcare systems globally [2].

In recent years, there has been a significant increase in the prevalence of chronic kidney disease (CKD). This condition has become a persistent health challenge, affecting populations across different continents and demographic groups. CKD, characterized by a gradual decline in kidney function, is associated with significant morbidity and mortality [3]. Its prevalence has been consistently rising on a global scale. The issue has gained significant recognition on a global scale as a prominent health concern, leading to collaborative endeavors aimed at minimizing its effects. Upon initial observation, these two conditions may seem separate, each with its causes, clinical symptoms, and treatment methods [4]. However, their intricate connection surpasses mere coincidence. The relationship between diabetes and kidney disease is bidirectional and multifaceted, showcasing an intricate network of connections encompassing pathophysiology, clinical management, and epidemiology. This complex interplay is essential for healthcare professionals, researchers, and policymakers [5].

One aspect of this intricate relationship involves acknowledging that diabetes is a significant risk factor for developing and advancing kidney disease. People diagnosed with diabetes have a significantly increased risk of developing CKD [6]. This highlights the importance of effectively managing diabetes to prevent complications related to the kidneys. Diabetic nephropathy, a distinct type of kidney disease closely linked to diabetes, significantly contributes to the increasing prevalence of end-stage renal disease (ESRD). On the other hand, kidney disease can contribute to the development of diabetes, thus emphasizing the reciprocal nature of this association. The kidneys are crucial in regulating glucose levels by filtering blood and selectively reabsorbing glucose as required. In the context of CKD, a decline in kidney function can give rise to compromised glucose metabolism, thereby fostering the development of insulin resistance and, ultimately, diabetes [6]. Therefore, managing kidney disease entails not only the preservation of renal function but also the prevention of diabetes in individuals who are susceptible to it [7].

Additionally, the coexistence of diabetes and kidney disease in a patient presents substantial clinical complexities. These comorbid conditions frequently require complex treatment plans that carefully manage glycemic control and preservation of kidney function [8]. Failure to consider this complex interaction can result in adverse health consequences, such as cardiovascular issues, heightened hospital admissions, and a reduced standard of living for those impacted. Considering the interconnectedness of these conditions and their significant implications for public health, clinical practice, and healthcare expenditures, it is crucial to investigate the reciprocal relationship between diabetes and kidney disease [7,8]. The primary goal of this narrative review is to address an essential objective by offering healthcare professionals, researchers, and policymakers a thorough and current comprehension of this complex relationship. The subsequent sections will provide a detailed explanation of the purpose and scope of this review. However, it is crucial to highlight the significant importance of this investigation. In addition to the inherent complexity of the relationship between diabetes and kidney disease, this two-way connection's ramifications extend beyond individual health outcomes [5-7]. These impacts resonate throughout healthcare systems, socioeconomic landscapes, and public health policy arenas, presenting a solid argument for a comprehensive analysis [8].

In the subsequent headings, we will thoroughly examine the complex network of interconnections. We will examine the pathophysiological mechanisms that connect diabetes and kidney disease, clarify the clinical manifestations and diagnostic methods, explore the various management approaches, discuss the critical role of nutrition, delve into pharmacological interventions, emphasize the significance of patient education and self-care, and highlight the current research directions. Through this comprehensive investigation, we aim to contribute to the more profound comprehension of this mutually influential connection. By doing so, we aim to facilitate well-informed clinical judgments, improve patient care, and enhance the public health response to the interconnected issues of diabetes and kidney disease.

## Review

Epidemiology of diabetes and kidney disease

Diabetes mellitus has become a significant global health issue, impacting a large number of individuals across the globe. Based on estimates provided by the International Diabetes Federation (IDF), it is reported that approximately 463 million adults between the ages of 20 and 79 were diagnosed with diabetes in the year 2019 [1]. It is worth noting that there are regional disparities in the prevalence of diabetes, with higher rates reported in the Western Pacific and Southeast Asia regions [2]. High-income countries exhibit a greater prevalence of diabetes than low- and middle-income countries. The increase in the prevalence of diabetes can be attributed to various factors, including sedentary lifestyles, unhealthy dietary habits, and rising obesity rates. These factors have become more widespread in contemporary society. In addition, it is worth noting that there is a changing age distribution in the occurrence of diabetes. There has been a notable rise in diagnoses among children and adolescents, indicating a noteworthy demographic shift [2,3].

CKD is a significant global health issue that affects approximately 10% of the world's population. The prevalence of CKD exhibits notable regional variations. It is subject to the influence of various factors, including but not limited to aging populations and the prevalence rates of diabetes and hypertension [4]. CKD is classified into stages, ranging from stage 1, the least severe, to stage 5, which indicates ESRD and requires either dialysis or kidney transplantation. Although the prevalence of CKD is considerable, the occurrence of its progression to ESRD is relatively infrequent [5]. However, it is essential to note that the development of ESRD is associated with substantial healthcare expenses and morbidity. It is worth noting that disadvantaged populations frequently experience a disproportionate CKD burden due to limited healthcare access, inadequate nutrition, and various other social determinants of health [6].

Significance of Diabetes as a Risk Factor for Kidney Disease

Diabetes is a significant risk factor for kidney disease, specifically in diabetic nephropathy. According to estimates, a significant percentage, ranging from 20% to 40%, of individuals diagnosed with diabetes are likely to develop diabetic nephropathy, highlighting its importance in clinical practice. The pathophysiology of diabetic nephropathy is characterized by the presence of chronic hyperglycemia, which results in progressive damage of the renal microvasculature [7]. This vascular impairment ultimately leads to compromised filtration and renal dysfunction. It is important to note that diabetic patients with inadequately controlled blood sugar levels and unmanaged hypertension face a significantly elevated risk of developing ESRD. Furthermore, a connection exists between diabetes and kidney disease that extends beyond the renal system [8]. This connection increases the likelihood of experiencing cardiovascular complications, including heart attacks and strokes. The complex nature of this relationship imposes a significant strain on healthcare systems, resulting in elevated healthcare expenses and requiring the implementation of comprehensive management strategies that include lifestyle adjustments, blood pressure regulation, and pharmaceutical interventions [9]. Understanding the importance of diabetes as a risk factor for kidney disease is paramount for healthcare professionals, researchers, and policymakers. This knowledge informs the development of strategies for early detection, prevention, and management of kidney disease. It also emphasizes the urgent requirement for public health initiatives focused on diabetes prevention and optimal care to address the increasing burden of kidney disease globally [10].

Complex pathophysiological interplay: understanding the mechanisms of kidney disease in diabetes

Diabetic kidney disease, or diabetic nephropathy, is a prevalent and debilitating complication of diabetes mellitus. It plays a significant role in the global burden of CKD [11]. It is of utmost importance to comprehend the mechanisms that underlie kidney disease in individuals with diabetes, as this knowledge is essential for effectively preventing and managing this intricate condition. This narrative review delves into the complex pathophysiological mechanisms that connect diabetes and kidney disease, emphasizing the crucial role played by hyperglycemia, inflammation, oxidative stress, and genetics.

Hyperglycemia: The Primary Cause

Hyperglycemia, characterized by elevated blood glucose levels, is recognized as the primary underlying factor contributing to the development and progression of kidney disease in individuals with diabetes. The kidneys can undergo a series of pathological changes, collectively known as diabetic nephropathy, due to prolonged exposure to elevated glucose levels [12]. One of the primary mechanisms involved in this process is activating the polyol pathway. This pathway metabolizes excess glucose into sorbitol, leading to cellular osmotic stress and oxidative damage. This process is known to contribute to dysfunction and injury of renal cells. In addition, it is worth noting that hyperglycemia has the potential to induce the synthesis of advanced glycation end-products (AGEs), leading to their accumulation in renal tissues [13]. AGEs can bind to renal cell receptors, activating pathways associated with inflammation and fibrosis. The persistent inflammation and fibrosis ultimately lead to renal damage and dysfunction [14].

Hyperglycemia is also implicated in the heightened activation of various isoforms of protein kinase C (PKC) in the kidney, resulting in modified cellular signaling and gene expression [15]. The activation of PKC plays a significant role in developing vascular dysfunction, heightened endothelial permeability, and the synthesis of extracellular matrix proteins [16]. These processes collectively contribute to the progression of kidney disease. In addition, it is essential to note that oxidative stress induced by hyperglycemia plays a significant role in developing kidney damage. Excessive glucose stimulates the generation of reactive oxygen species (ROS), which can overpower the body's antioxidant defense mechanisms. The subsequent occurrence of oxidative stress induces lipid peroxidation, DNA damage, and inflammation within the renal tissues [16].

Role of Inflammation

Inflammation is a characteristic feature of diabetic nephropathy and is closely associated with hyperglycemia. Increased glucose levels can initiate multiple inflammatory pathways, such as the activation of nuclear factor-kappa B (NF-κB) and the generation of pro-inflammatory cytokines. In response to chemotactic signals, inflammatory cells, including macrophages, infiltrate the renal tissue [17]. These immune cells secrete inflammatory mediators, which contribute to the ongoing damage and fibrosis of the tissue. In addition, the presence of immune cells has been linked to the emergence of microalbuminuria, which serves as an early indicator of kidney dysfunction in individuals with diabetes [15-17]. In addition, inflammation can impair endothelial function, negatively affecting renal blood flow regulation. The compromised blood flow worsens the condition of inadequate oxygen supply to the kidneys, which is a crucial element in the progression of diabetic nephropathy. Kidney inflammation can worsen hyperglycemia, creating a detrimental cycle [18]. Inflammatory cytokines, such as tumor necrosis factor-alpha (TNF-α) and interleukin-6 (IL-6), can potentially hinder insulin signaling in renal cells, resulting in insulin resistance. Insulin resistance can potentially exacerbate glycemic control, thus perpetuating the inflammatory response caused by hyperglycemia [19].

Oxidative Stress: A Molecular Phenomenon

Oxidative stress, which occurs due to an imbalance between the production of ROS and the body's antioxidant defenses, plays a significant role in developing diabetic kidney disease. Hyperglycemia is a significant catalyst for oxidative stress, resulting in the excessive production of ROS in renal tissues [20]. Excessive ROS can lead to oxidative damage in renal cells, affecting lipids, proteins, and DNA. Lipid peroxidation leads to the generation of harmful byproducts, worsening tissue damage. The occurrence of oxidative damage to DNA can activate signaling pathways associated with inflammation and fibrosis [21]. Additionally, it is essential to note that oxidative stress has the potential to affect the function and integrity of podocytes directly. Podocytes are a distinct type of cells within the kidney's filtration barrier. Their impaired function significantly develops proteinuria, a characteristic feature of diabetic nephropathy [22]. Furthermore, oxidative stress can potentially enhance inflammation by facilitating the secretion of pro-inflammatory cytokines and the initiation of NF-κB signaling pathways. The interaction between oxidative stress and inflammation establishes a detrimental cycle that propels the advancement of kidney disease in individuals with diabetes [23].

Genetic Susceptibility: Deciphering the Genetic Factors

Hyperglycemia, inflammation, and oxidative stress are significant factors contributing to the development of diabetic nephropathy. However, it is essential to acknowledge that genetics also substantially influence an individual's vulnerability to kidney disease in the context of diabetes [24]. Numerous genetic factors have been associated with the onset and advancement of diabetic kidney disease. For example, specific polymorphisms in genes associated with glucose metabolism, such as the ACE gene, have been linked to an elevated susceptibility to diabetic nephropathy. There is a correlation between genetic variations in the renin-angiotensin-aldosterone system (RAAS) and susceptibility to kidney disease [25].

In addition, it is worth noting that genetics can play a significant role in determining an individual's response to therapeutic interventions. The presence of genetic variations can potentially influence how individuals metabolize medications that are prescribed for the management of diabetes and kidney disease. This, in turn, can impact the effectiveness and safety of the treatment provided [26]. In summary, it is imperative to comprehend the mechanisms that drive kidney disease in individuals with diabetes to formulate efficient prevention and management strategies. Hyperglycemia is considered the primary cause, triggering events that ultimately result in renal dysfunction. The presence of inflammation, oxidative stress, and genetic factors significantly contribute to the worsening of kidney injury in cases of diabetic nephropathy [27]. This intricate interaction highlights the need for personalized strategies to prevent and treat this condition. The progress made in comprehending these mechanisms has the potential to enhance outcomes and improve the quality of life for individuals affected by diabetes and kidney disease [28].

Interplay between kidney disease and diabetes: revealing underlying mechanisms

The relationship between kidney disease and diabetes is complex, encompassing more than just diabetes as a risk factor for kidney disease. In an underexplored yet equally consequential dimension, kidney disease has the potential to induce or worsen diabetes [29]. This narrative review explores the underlying mechanisms by which kidney disease, specifically CKD and diabetic nephropathy, may contribute to the onset or progression of diabetes. The text also explores the concept of diabetic nephropathy and its complex influence on glucose metabolism [30].

Insulin Resistance: A Key Mechanism

The disruption of insulin signaling can occur due to kidney disease, particularly in its advanced stages. This can lead to the development of insulin resistance, which is a characteristic feature of type 2 diabetes [30]. Insulin resistance is a physiological condition characterized by reduced responsiveness of cells in the body to the effects of insulin. In individuals with compromised renal function, the signaling pathways of insulin may experience disruption due to various factors, including inflammation and oxidative stress [30]. The compromised signaling hinders insulin's capacity to effectively facilitate glucose uptake in peripheral tissues, leading to hyperglycemia, a prominent feature of diabetes. In kidney disease, insulin resistance can lead to diminished sensitivity of peripheral tissues, including muscle and adipose cells, towards insulin. Consequently, these tissues experience a decrease in their efficiency in the uptake and utilization of glucose from the bloodstream [30]. The decrease in glucose uptake worsens hyperglycemia, as the glucose level remains elevated in the bloodstream.

Dysregulated Glucose Handling in Renal Disease

Impairment of renal glucose reabsorption: The kidneys are critical in regulating glucose levels by filtering the blood and selectively reabsorbing glucose to maintain glucose homeostasis. However, in CKD, the intricate regulation of glucose handling may become compromised [4-6]. Renal glucose reabsorption deficiencies can result in glucosuria, a medical condition characterized by detecting glucose in the urine. This phenomenon contributes to increased blood glucose levels, bringing the individual closer to a state of diabetes. The metabolic disruptions linked to CKD can significantly impact glucose metabolism. Patients diagnosed with CKD may present with atypical glucose metabolism patterns, such as impaired glucose tolerance and diminished insulin sensitivity [3-7]. The modifications in glucose metabolism can potentially elevate the likelihood of developing diabetes.

Inflammation and Oxidative Stress: Contributing to the Diabetic Cascade

Chronic inflammation: Kidney disease is frequently accompanied by chronic inflammation, primarily driven by factors such as immune cell infiltration and the release of pro-inflammatory cytokines. This inflammatory environment contributes to the occurrence of insulin resistance, which is a crucial factor in the progression of diabetes [8]. The presence of pro-inflammatory cytokines has the potential to disrupt insulin signaling, resulting in compromised glucose uptake and utilization in peripheral tissues. Oxidative stress is a significant factor in kidney disease, marked by an imbalance between the generation of ROS and the body's antioxidant mechanisms. Hyperglycemia, a prevalent characteristic of diabetes and diabetic nephropathy, is a significant catalyst for oxidative stress [10]. The overproduction of ROS in renal tissues can result in oxidative damage to lipids, proteins, and DNA. The oxidative damage not only plays a role in the development of kidney injury but also hinders the proper functioning of insulin signaling pathways, thereby worsening insulin resistance [11].

Diabetic Nephropathy: Understanding the Multifaceted Effects on Glucose Metabolism

Diabetic nephropathy, a distinct type of renal disease closely associated with diabetes, plays a significant role in the complex interplay between kidney disease and diabetes [15]. The condition is characterized by notable structural and functional alterations in the kidney, primarily resulting from prolonged exposure to elevated glucose levels in the bloodstream. It is essential to have a comprehensive understanding of the influence of diabetic nephropathy on glucose metabolism to grasp the potential contribution of kidney disease to diabetes [17].

Renal Glucose Reabsorption: Implications for Hyperglycemia

Renal glucose handling in healthy individuals: In individuals with normal kidney function, the vast majority of filtered glucose is effectively reabsorbed by renal tubules and reintroduced into the bloodstream. This mechanism conserves glucose, thereby preventing its excretion in the urine [18]. The glucose-handling process becomes impaired in the context of diabetic nephropathy. Glucosuria can occur when the renal tubules experience a decline in their capacity to reabsorb glucose efficiently. Consequently, individuals diagnosed with diabetic nephropathy may encounter chronic hyperglycemia due to the excretion of glucose in the urine instead of its retention in the bloodstream [20].

Insulin Clearance and Hormonal Dysregulation: Factors Affecting Glucose Control

The process of renal insulin clearance involves the kidneys' contribution to removing insulin from the circulatory system. Impaired renal function, as observed in diabetic nephropathy, may result in decreased insulin clearance. This leads to elevated insulin levels in the bloodstream, which initially counteracts insulin resistance by facilitating glucose absorption. Nevertheless, the extended elevation of insulin levels may lead to the depletion of pancreatic beta cells, ultimately contributing to the onset of diabetes [21]. Kidney disease can disturb the equilibrium of multiple hormones, including those that affect glucose metabolism. An instance of this is when diabetic nephropathy causes changes in the RAAS, which impacts blood pressure and glucose homeostasis regulation. Moreover, hormonal fluctuations associated with kidney disease, such as modifications in erythropoietin and fibroblast growth factor-23 (FGF-23) concentrations, can impact glucose regulation [22].

Inflammation, Fibrosis, and Insulin Resistance: An Intricate Interplay

Diabetic nephropathy is distinguished by chronic inflammation and the accumulation of extracellular matrix proteins within renal tissues, leading to inflammation and fibrosis. The aforementioned pathophysiological changes play a role in advancing renal damage [12]. However, these findings also have significant implications for glucose metabolism. The release of inflammatory mediators triggered by kidney injury has the potential to disrupt insulin signaling pathways, resulting in the development of insulin resistance [13].

Clinical presentation and diagnostic approaches for renal disease in patients with diabetes

CKD is a commonly occurring and debilitating complication of diabetes mellitus, which has the potential to significantly affect multiple facets of an individual's health and overall quality of life. It is essential to have a comprehensive understanding of the clinical manifestations and diagnostic techniques for kidney disease in individuals with diabetes [14]. This knowledge is vital for early detection, efficient management, and enhanced patient outcomes. This narrative review offers valuable insights into the clinical presentation of kidney disease in individuals with diabetes. It examines the diagnostic methods employed to evaluate kidney function in this high-risk population [15].

Clinical manifestations of kidney disease in diabetes

During the initial phases of kidney disease, individuals with diabetes may exhibit no noticeable symptoms. It is imperative to conduct regular screening for kidney function in this particular population, as the early identification of any abnormalities enables prompt intervention to mitigate the progression of the disease effectively [16]. Microalbuminuria is characterized by small amounts of albumin in the urine, typically 30-300 mg daily. It is commonly observed as one of the initial indicators of kidney impairment in individuals with diabetes. Although it may not manifest with overt symptoms, it is a pivotal indicator of renal impairment [17].

In the course of kidney disease advancement, individuals may experience the development of macroalbuminuria or proteinuria, which is characterized by elevated levels of protein in the urine. This may present as urine with a frothy or foamy appearance. Proteinuria may also be correlated with edema, specifically in the lower extremities, such as the legs and ankles [20]. Hypertension, or high blood pressure, is a comorbidity in individuals with diabetes and kidney disease. Kidney disease can contribute to the development of elevated blood pressure levels. Hypertension can worsen kidney damage and elevate the likelihood of cardiovascular complications [1-5]. The glomerular filtration rate (GFR) decreases as kidney function declines, reducing GFR. The decrease in GFR can lead to elevated serum creatinine levels, a decrease in estimated GFR (eGFR), and changes in electrolyte equilibrium [9].

Fatigue, weakness, and decreased cognitive function may manifest as kidney disease progresses. The presence of non-specific symptoms can have a detrimental effect on an individual's overall quality of life. Anemia can occur due to kidney disease, resulting in symptoms such as fatigue, pallor, and decreased exercise capacity [10]. Anemia in individuals with diabetes-related kidney disease is frequently associated with a reduction in the production of erythropoietin by the kidneys. Fluid and electrolyte imbalances can occur due to kidney dysfunction, which can interfere with the body's ability to regulate and maintain proper fluid and electrolyte levels [12]. These potential outcomes include the development of edema, hypertension, electrolyte imbalances such as hyperkalemia, and metabolic acidosis. Kidney disease can disrupt calcium and phosphate metabolism, resulting in possible complications such as bone pain and fractures. These disruptions may also contribute to the development of secondary hyperparathyroidism [14].

Diagnostic Methods for Evaluating Renal Function in Patients with Diabetes

Serum creatinine levels are frequently utilized as a means to estimate renal function. An increased serum creatinine indicates a reduced GFR. Nevertheless, it is essential to note that creatinine levels may continue to fall within the established normal range until substantial kidney function impairment has transpired [16]. The eGFR is determined through the utilization of different equations, such as the Modification of Diet in Renal Disease (MDRD) and Chronic Kidney Disease Epidemiology Collaboration (CKD-EPI) equations [17]. By incorporating these equations, eGFR offers a more precise kidney function assessment than solely on creatinine levels. An eGFR below 60 mL/min/1.73 m² suggests CKD [20].

The urine albumin-to-creatinine ratio (UACR) is a diagnostic tool that quantifies the albumin level in urine concerning creatinine. It is commonly employed for the detection and ongoing monitoring of proteinuria. This tool is precious for the early detection of kidney damage [22]. The renal ultrasound is a non-invasive imaging modality to evaluate kidney dimensions, morphology, and structural anomalies. This diagnostic procedure can assist in identifying various conditions, including hydronephrosis, cysts, or renal artery stenosis, which can contribute to kidney dysfunction. A kidney biopsy may be required in certain instances to ascertain the root cause of kidney disease and guide treatment decisions [23]. However, this approach is generally utilized in cases where the diagnosis remains uncertain or specific interventions are deemed necessary [24].

It is crucial to monitor serum electrolyte levels, such as potassium, calcium, and phosphate, in individuals with kidney disease and assess their acid-base status. This tool aids in the identification and management of electrolyte imbalances, as well as the assessment of metabolic acidosis [15]. Consistent blood pressure monitoring is essential for individuals diagnosed with diabetes and kidney disease. Hypertension is a prevalent comorbidity requiring meticulous management to mitigate the risk of additional renal impairment. Assessing hemoglobin and hematocrit levels is crucial in diagnosing and monitoring anemia, a prevalent complication of kidney disease [17]. The management of anemia may involve using erythropoiesis-stimulating agents and iron supplementation. Bone Density Testing: Individuals with advanced kidney disease may undergo bone density testing, such as dual-energy X-ray absorptiometry (DEXA), to evaluate bone health and identify mineral and bone disorders linked to CKD [20].

Identifying and assessing clinical symptoms and diagnostic techniques for kidney disease in individuals with diabetes is essential for early detection and efficient management [12]. Identifying subtle indicators of kidney involvement, such as microalbuminuria and hypertension, is crucial to implementing necessary interventions promptly [14]. Various diagnostic tools, such as eGFR, UACR, renal ultrasound, and laboratory tests, are crucial in evaluating kidney function and informing treatment approaches. Consistent monitoring and implementing a multidisciplinary approach are essential to maximize outcomes and minimize the effects of kidney disease on the overall health and quality of life of individuals with diabetes [15].

Management strategies for kidney disease in diabetes

The optimal management of kidney disease in individuals with diabetes necessitates a comprehensive strategy encompassing various aspects, including prevention, early detection, and therapeutic interventions. This narrative review examines the strategies for preventing kidney disease in individuals with diabetes [10]. It offers insights into therapeutic interventions, encompassing lifestyle modifications, pharmacological treatments, and surgical options [9].

Strategies for Mitigating Kidney Disease in Individuals Affected by Diabetes

Implementing preventive measures is a fundamental aspect of effectively managing kidney disease in individuals with diabetes, with the primary objective of delaying or potentially stopping its advancement [22]. Implementing the following strategies is crucial for preventing kidney disease in this population at high risk.

Lifestyle modifications: Lifestyle interventions are essential in preventing kidney disease among individuals with diabetes. These factors encompass attaining and sustaining optimal blood glucose control through a balanced diet, consistent engagement in physical activity, and effective weight management [23]. Managing blood pressure is equally important, with implementing dietary sodium restriction and utilizing antihypertensive medications as essential components. It is imperative to prioritize smoking cessation, as smoking can potentially worsen kidney damage [24].

Pharmacological treatments: Medications can potentially mitigate the risk of kidney disease in individuals diagnosed with diabetes. Angiotensin-converting enzyme (ACE) inhibitors and angiotensin receptor blockers (ARBs) are commonly prescribed to individuals diagnosed with diabetes and hypertension to mitigate proteinuria and decelerate the advancement of kidney disease [25]. Furthermore, it has been observed that sodium-glucose cotransporter-2 (SGLT-2) inhibitors exhibit the potential to preserve kidney function and mitigate cardiovascular events among individuals with diabetes. Proper management of dyslipidemia and glycemic control using medications can also significantly prevent kidney disease [26].

Routine monitoring: It is essential to regularly monitor kidney function by assessing serum creatinine levels, eGFR, and UACR to identify any issues and provide appropriate intervention promptly [4]. This enables healthcare providers to detect kidney disease in its initial stages and implement suitable interventions to decelerate its advancement [5].

Blood pressure management: Hypertension frequently coexists as a comorbidity in individuals diagnosed with diabetes and kidney disease. Implementing a proactive approach to blood pressure management is crucial, with a specific focus on achieving a target of less than 130/80 mm Hg [6]. This approach is vital in mitigating the potential risk of kidney damage. To achieve and sustain optimal blood pressure levels, it may be necessary to implement a combination of lifestyle modifications and antihypertensive medications [7].

Therapeutic Interventions for the Management of Diabetic Kidney Disease

When kidney disease is already present, it is crucial to implement a comprehensive therapeutic approach. Management strategies include lifestyle modifications, pharmacological treatments, and surgical options to effectively address the specific challenges associated with kidney disease in individuals with diabetes [26].

Lifestyle modifications: Implementing lifestyle changes remains fundamental to managing kidney disease among individuals with diabetes. Implementing dietary modifications, such as limiting sodium intake and adjusting protein consumption, can effectively facilitate the regulation of fluid balance and alleviate the strain on renal function [27]. In advanced stages of kidney disease, it may also be necessary to restrict the intake of potassium and phosphate. In addition, individuals with kidney disease should restrict their alcohol intake and refrain from smoking to mitigate the risk of exacerbating kidney damage.

Pharmacological treatments: These encompass a range of interventions designed to effectively mitigate the progression of kidney disease and effectively address its associated complications [28]. ACE inhibitors and ARBs remain the preferred initial therapies for reducing proteinuria and blood pressure. SGLT-2 inhibitors have shown efficacy beyond their preventive function, exhibiting positive effects in decelerating kidney disease progression and reducing cardiovascular events in individuals with pre-existing kidney disease and diabetes [29]. In addition, a comprehensive treatment plan may include prescribing medications such as statins and erythropoiesis-stimulating agents to manage dyslipidemia and anemia, respectively [30]. Achieving optimal blood glucose control is imperative for effectively managing kidney disease in individuals diagnosed with diabetes. It may be necessary to personalize glycemic management targets, considering various factors such as age, comorbidities, and the potential risk of hypoglycemia [24]. Continuous glucose monitoring (CGM) systems offer significant benefits by providing valuable insights into blood glucose trends and aiding in achieving optimal glycemic control. Blood pressure management is an essential component of effectively managing kidney disease. To attain desired blood pressure levels, it may be necessary to utilize a combination of antihypertensive medications, such as ACE inhibitors, ARBs, diuretics, and calcium channel blockers [25]. Consistent monitoring of blood pressure and making necessary adjustments to the treatment regimen are essential for maintaining optimal control.

Surgical interventions: Surgery may be considered in cases of advanced kidney disease. Kidney transplantation is widely regarded as the most efficacious long-term treatment option for individuals with ESRD. It has been shown to enhance quality of life significantly. Living donor kidney transplantation is a feasible alternative, and advancements in surgical techniques have significantly enhanced the overall results [26]. Hemodialysis and peritoneal dialysis are viable alternatives for individuals awaiting transplantation or for whom transplantation is not viable. The surgical placement of vascular access for hemodialysis, such as arteriovenous fistulas or grafts, may be required [27].

The effective management of kidney disease in individuals with diabetes requires a comprehensive approach encompassing prevention, early detection, and therapeutic interventions. Preventive strategies involve implementing lifestyle modifications, utilizing pharmacological treatments, and maintaining regular monitoring to mitigate the risk of kidney disease [28]. In cases where kidney disease is already present, adopting a multidisciplinary approach that encompasses lifestyle modifications, pharmacological interventions, and, when necessary, surgical interventions is imperative. This comprehensive approach aims to decelerate the progression of the disease and enhance the overall quality of life [29]. Healthcare providers can enhance patient outcomes and alleviate the burden of kidney disease in individuals with diabetes by addressing the distinct challenges associated with this condition.

Nutritional considerations for the management of diabetes and kidney disease

Ensuring adequate nutrition is essential for effectively managing both diabetes and kidney disease. This narrative review examines the crucial role of diet in managing these concurrent conditions, discussing dietary limitations, protein consumption, and the influence of different nutrients. The role of diet in managing diabetes and kidney disease is of utmost importance, as it significantly impacts blood glucose levels, blood pressure, and overall kidney function. Following a well-balanced diet customized to meet individual needs can assist individuals in attaining enhanced glycemic control, impeding the advancement of kidney disease and enhancing overall health [30]. Managing dietary restrictions is crucial in effectively addressing the challenges of diabetes and kidney disease. It is imperative to adhere to a sodium-restricted diet to manage hypertension and maintain proper fluid balance effectively. Consuming excessive amounts of sodium can result in fluid retention and elevated blood pressure [30]. It is essential for individuals with diabetes and kidney disease, particularly in advanced stages of kidney disease, to monitor their potassium and phosphorus intake closely. This is because these electrolytes have the potential to accumulate in the bloodstream and cause imbalances [15]. In addition, it is imperative to restrict alcohol consumption and refrain from using tobacco products to promote optimal health and effectively manage both conditions.

Protein Consumption

The consideration of protein intake is of utmost importance in the dietary management of individuals with diabetes and kidney disease. Protein restriction is frequently advised to alleviate the strain on the kidneys, especially during the advanced stages of kidney disease [16]. Reducing the consumption of high-protein foods, such as red meat and processed meats, can effectively mitigate the generation of waste products that necessitate kidney filtration. Nevertheless, finding a harmonious equilibrium between diminishing protein consumption and guaranteeing sufficient nutritional intake is crucial [17]. Individuals should seek guidance from a registered dietitian to ascertain their precise protein requirements, considering factors such as their stage of kidney disease, nutritional status, and level of physical activity [18].

Effects of Different Nutrients

Carbohydrates play a crucial role in the management of diabetes, as monitoring carbohydrate intake and effectively managing glycemic control are critical aspects of diabetes management. Carbohydrates have a notable impact on blood glucose levels, and individuals with diabetes should prioritize consuming complex carbohydrates with a low glycemic index [19]. Consuming foods high in fiber, such as whole grains, fruits, and vegetables, can contribute to the stabilization of blood sugar levels and enhance feelings of satiety. Dietary fats have the potential to impact lipid profiles and cardiovascular health, which is a matter of considerable importance for individuals who have diabetes and kidney disease [20]. Emphasizing the consumption of healthy fats, specifically unsaturated fats present in olive oil, nuts, and fatty fish, has been shown to enhance lipid profiles and mitigate the likelihood of cardiovascular complications. Ensuring sufficient intake of vitamins and minerals is essential for maintaining optimal health. Vitamin D and calcium are crucial in preserving bone health, especially for individuals diagnosed with kidney disease. Vitamin D supplementation may be required in cases where blood levels indicate deficiency [22]. Furthermore, individuals diagnosed with kidney disease may necessitate modifications in their calcium, phosphorus, and potassium consumption due to their unique dietary limitations and medical treatment [24].

*Fluid Intake*****

Adequate fluid management is crucial for individuals diagnosed with kidney disease, particularly those undergoing hemodialysis or peritoneal dialysis. It may be necessary to limit fluid intake to prevent the occurrence of fluid overload and imbalances in electrolyte levels [25]. Regularly monitoring urine output and seeking guidance from a healthcare provider or dietitian can assist in determining suitable fluid allowances. In summary, nutrition is crucial in managing diabetes and kidney disease. It is crucial to consider dietary restrictions, protein intake, and the effects of different nutrients to effectively manage glycemic control, mitigate the advancement of kidney disease, and enhance overall health [26]. Customizing dietary recommendations to meet individual needs, closely monitoring nutrient intake, and seeking guidance from healthcare professionals and registered dietitians are crucial components in effectively managing the dietary needs of individuals with these concurrent conditions [28].

Pharmacological therapies for the management of diabetes and kidney disease

Pharmacological interventions play a vital role in managing diabetes and kidney disease, aiding individuals in achieving glycemic control, regulating blood pressure, and mitigating the advancement of kidney damage. This narrative review examines medications frequently employed in the management of these concurrent conditions, discusses potential drug interactions, and provides insights into considerations for medication adjustments. The successful management of diabetes and kidney disease often requires a personalized combination of medications [24]. Antidiabetic agents are often prescribed to individuals with diabetes to manage their blood sugar levels effectively. These medications belong to different classes and are chosen based on each patient's needs to achieve optimal glycemic control. The oral agents used in the treatment of diabetes include metformin, sulfonylureas, dipeptidyl peptidase-4 (DPP-4) inhibitors, SGLT-2 inhibitors, and glucagon-like peptide-1 (GLP-1) receptor agonists [25]. Each class of medication possesses distinct mechanisms of action and potential advantages in managing diabetes [30].

Antihypertensive Medications

Hypertension alongside diabetes and kidney disease necessitates prioritizing blood pressure control. Commonly prescribed medications for hypertension include ACE inhibitors, ARBs, beta-blockers, diuretics, calcium channel blockers, and alpha-blockers. ACE inhibitors and angiotensin receptor blockers ARBs are known for their renoprotective properties [22]. They are frequently prescribed to patients diagnosed with diabetic nephropathy. Lipid-lowering agents are commonly prescribed to individuals with diabetes and kidney disease due to the high prevalence of dyslipidemia, significantly increasing the likelihood of developing cardiovascular complications. Statins are the predominant pharmacological agents to manage lipid levels and mitigate cardiovascular risk [24]. In certain instances, it may be necessary to consider combination therapy with additional agents, such as ezetimibe or fibrates.

Medications for Anemia

Anemia frequently arises as a complication of kidney disease. To address this, erythropoiesis-stimulating agents (ESAs), such as erythropoietin analogs, are employed to stimulate the production of red blood cells and effectively manage anemia [25]. Iron supplementation may be prescribed as a treatment for iron deficiency anemia. Phosphate binders are commonly used in cases of advanced kidney disease to address the occurrence of hyperphosphatemia, which arises from the kidneys' reduced ability to excrete phosphate. Phosphate binders, such as calcium-based or non-calcium-based binders like sevelamer, mitigate phosphate absorption from dietary sources and uphold serum phosphate levels within desired thresholds [26].

Exploring potential drug interactions and factors to consider for medication adjustments

Effectively managing pharmacological interventions in individuals with diabetes and kidney disease necessitates careful attention to potential drug interactions and the need for dosage adjustments based on renal function [27].

Drug Interactions

It is common for individuals with diabetes and kidney disease to be prescribed multiple medications, elevating the likelihood of drug interactions. Evaluating potential interactions among antidiabetic agents, antihypertensive medications, lipid-lowering agents, and other pharmaceuticals is essential [28]. For example, certain medications can enhance the hypoglycemic effects of antidiabetic agents, thereby increasing the likelihood of experiencing hypoglycemia. Close monitoring and adjusting medication may be necessary to mitigate these risks [29].

Renal Function

The presence of kidney disease has a substantial impact on drug clearance and metabolism. Medications primarily eliminated through renal excretion may necessitate dosage modifications or alternative treatment options in individuals with impaired renal function [12]. For example, it is worth noting that metformin, a frequently prescribed antidiabetic medication, can accumulate in the bloodstream when kidney dysfunction is present, potentially resulting in lactic acidosis [13]. Therefore, it is frequently not recommended for individuals with severe kidney disease. It is crucial to consider the necessary adjustments in the dosages of medications cleared by the kidneys and the selection of alternative agents that have minimal excretion through the renal system [14].

Monitoring and Collaboration

It is of utmost importance to regularly monitor renal function, blood pressure, glycemic control, and medication adherence in individuals with diabetes and kidney disease. Implementing collaborative care involving healthcare providers, pharmacists, and nephrologists is crucial to optimizing medication regimens for the dual purposes of glycemic control and kidney health [24]. Medication adjustments based on individual patient profiles are recommended, considering comorbidities, renal function, and potential drug interactions. In summary, pharmacological interventions are crucial in managing diabetes and kidney disease. Implementing a customized strategy that includes antidiabetic agents, antihypertensive medications, lipid-lowering agents, and drugs for anemia and hyperphosphatemia is crucial to attain the best possible results [15]. Healthcare providers must maintain a high vigilance when evaluating potential drug interactions and implementing appropriate medication modifications following renal function and each patient's specific needs. The implementation of a collaborative and multidisciplinary approach is crucial to ensure that medication regimens effectively manage both diabetes and kidney disease while also prioritizing safety and tolerability [16].

Current research and future perspectives on the reciprocal relationship between diabetes and kidney disease

As the bidirectional link between diabetes and kidney disease undergoes further exploration, current research trends show potential for enhancing prevention and management strategies [17]. This narrative review aims to provide a comprehensive overview of the current research directions, potential breakthroughs, and areas requiring further investigation. It aims to enhance our understanding and improve outcomes for individuals affected by these interconnected conditions [18].

Emerging Research Trends and Prospective Breakthroughs

The current trend in research is centered around personalized approaches to managing diabetes and kidney disease, commonly referred to as precision medicine approaches. Researchers strive to identify personalized treatment strategies by integrating genetic, genomic, and clinical data [20]. Precision medicine can forecast an individual's susceptibility to kidney disease within the framework of diabetes and customize interventions based on their distinct genetic and physiological characteristics. Researchers are currently engaged in the exploration of novel biomarkers that have the potential to assist in the early detection and monitoring of kidney disease in individuals who have diabetes. In addition to conventional markers such as serum creatinine and urinary albumin, research is ongoing into novel biomarkers like kidney injury molecule-1 (KIM-1), neutrophil gelatinase-associated lipocalin (NGAL), and urinary proteomics [22]. These biomarkers hold promise in offering valuable insights into early-stage kidney damage.

Advanced imaging techniques: The field of imaging technologies is continuously advancing, with magnetic resonance imaging (MRI), computed tomography (CT), and contrast-enhanced ultrasound playing a significant role in improving the non-invasive evaluation of kidney structure and function [25]. These advanced imaging modalities provide enhanced accuracy in detecting kidney abnormalities and vascular changes linked to kidney disease related to diabetes. This study targets inflammation and fibrosis, recognized as significant contributors to kidney damage in individuals with diabetes [26]. Ongoing research is investigating innovative therapeutic targets to mitigate these biological processes effectively. Potential advancements include developing anti-inflammatory agents, antifibrotic medications, and novel methods for modulating the immune response, such as cytokines-based therapies.

Potential Areas for Further Research to Enhance Prevention and Management

Identifying reliable early biomarkers that can predict an individual's vulnerability to developing kidney disease in the context of diabetes continues to be a top priority [26]. Early risk stratification allows for targeted interventions, such as lifestyle modifications and pharmacological treatments, when interventions have the most significant impact. There is a pressing need for advancements in diagnostic techniques to evaluate the progression of kidney disease more effectively. The primary objective of the research should be to prioritize the development of tools capable of accurately detecting subtle changes in kidney function, fibrosis, and inflammation [28]. These tools will play a crucial role in guiding treatment adjustments and assessing the effectiveness of therapeutic interventions.

Investigating innovative therapeutic approaches: Although existing therapeutic options for managing kidney disease in diabetes have demonstrated potential, it is crucial to continue research efforts to explore novel therapies. This includes investigating regenerative medicine approaches, gene therapy and targeted molecular interventions [27]. Exploring emerging pharmacological agents, such as endothelin receptor antagonists and antifibrotic drugs, presents significant opportunities for investigation. Further research should be conducted to explore integrated care models that incorporate multidisciplinary teams consisting of nephrologists, endocrinologists, dietitians, and mental health professionals [28]. The implementation of integrated care has the potential to improve coordination, optimize medication management, and address psychosocial factors that can influence treatment adherence and overall well-being. Integrating patient-reported outcomes, including assessments of quality of life and patient-reported symptoms, is of utmost importance in research and clinical practice [29]. Gaining insight into the firsthand experiences of individuals affected by diabetes-related kidney disease can provide valuable knowledge for developing customized interventions and enhancing patient-centered care.

In summary, the current research on the reciprocal relationship between diabetes and kidney disease presents promising prospects for enhanced prevention and management approaches in the future [23]. Precision medicine, emerging biomarkers, state-of-the-art imaging techniques, and targeted therapies are facilitating the development of increasingly individualized and efficacious care strategies. However, significant areas still require additional research [30]. These areas include early risk stratification, enhanced diagnostic methods, innovative therapy investigation, integrated care model development, and a greater emphasis on patient-reported outcomes. The progress made in these fields holds the promise of revolutionizing the management of diabetes and kidney disease, thereby improving the overall well-being of individuals impacted by these interconnected conditions [30].

## Conclusions

In conclusion, this narrative review has provided an overview of the complex interplay between diabetes and kidney disease, underscoring its significant ramifications for the healthcare sector. The bidirectional relationship between these conditions is indisputable, as diabetes increases the likelihood of developing kidney disease. In contrast, kidney disease intensifies complications associated with diabetes. The findings obtained from this review reinforce the pressing requirement for comprehensive management strategies that consider the distinct challenges presented by this interplay. Understanding the importance of bidirectional links is crucial for healthcare professionals and policymakers alike. Implementing early detection, personalized treatment plans, lifestyle interventions, and collaborative care is crucial in enhancing patient outcomes. In addition, our efforts to tackle this intricate health challenge must be guided by continuous research, innovation, and a patient-centered approach. By adopting these recommendations, we can successfully mitigate the impact of diabetes and kidney disease, improve patient well-being, and establish a more robust healthcare system that can effectively address these interconnected conditions on a global level.
